# Community perspectives on drug promotion on social media during the COVID-19 pandemic in KwaZulu-Natal, South Africa

**DOI:** 10.12688/f1000research.158535.1

**Published:** 2025-01-06

**Authors:** Rujeko Samanthia Chimukuche, Julia Ndlazi, Janet Seeley

**Affiliations:** 1 Social Science Core, Africa Health Research Institute, Durban, KwaZulu-Natal, South Africa; 2 Division of Infection & Immunity, University College London, London, UK; 3 University of KwaZulu-Natal School of Nursing and Public Health, Durban, KwaZulu-Natal, South Africa; 4 Department of Global Health and Development, London School of Tropical Hygiene and Medicine, London, UK

**Keywords:** Drug promotion, drug advertising, social media, Ethics and COVID-19, traditional medicine

## Abstract

**Background:**

During the COVID-19 pandemic, social media and web-based platforms were widely used to promote medicinal substances. To assess community perspectives on drug promotions on social media, we conducted qualitative research using three workshops. The workshops aimed to highlight the public understanding of drug advertising focusing on community perceptions of social media drug promotions, their risks and benefits. Discussions were conducted on the importance of adhering to national drug regulation policies and the World Health Organisation ethical criteria for promotion, advertisement, and publicity of medicines.

**Methods:**

Participants for the workshops were purposively sampled from local community youth groups and healthcare facilities. Two workshops included ten young adults aged 18-35, while the third workshop involved three healthcare professionals and one traditional healer.

**Results:**

The study participants’ highlighted the value of honesty and trust in the drug promotions. Gaps in the ethical conduct of advertising were observed and concerns were raised about the reliability of social media information and the omission of valuable details on the drug advertisements.

**Conclusion:**

Individuals have a right to informed choices that ensure their health safety. This study has highlighted the need for transparency and accountability in pharmaceutical and complementary medicine marketing on social media. Collaboration is needed between regulatory bodies, pharmaceutical companies, healthcare providers and community members, to make sure that drug advertising upholds ethical standards and public health.

**Abbreviation**
COVID-19: Coronavirus disease 2019

## Background

The South African Health Products Regulatory guidelines state that all medicinal drug advertisements should promote registered products which are “ *accurate, complete, clear and designed to promote credibility and trust by the general public and health practitioners. Statements or illustrations must not mislead directly or by implication”.* ^1^ During the COVID-19 pandemic, several drug advertisements appeared on social media promoting treatments for COVID-19. We held workshops with young adults and selected health care providers in KwaZulu-Natal, South Africa to gather their perspectives on this advertising during the pandemic.

Before the pandemic, public health authorities had already utilized social media platforms to disseminate public health messages. ^2^ This approach allowed them to reach a wide audience quickly and effectively. ^3, 4^ However, the onset of the COVID-19 pandemic significantly accelerated this trend. ^5^ The urgency and global impact of the pandemic necessitated more immediate and widespread communication and social media became an essential tool for authorities to provide real-time updates, share preventive measures, clarify misinformation, and engage with the public on a more interactive level. ^6^

In Africa, in line with global trends, social media became the tool to share treatment suggestions including videos and audios of individuals, including traditional healers, claiming that herbs (including cannabis) can cure COVID-19. ^7, 8^ WhatsApp and X (Twitter) became online marketplaces for vending herbal treatments. ^9, 10^

In this paper we present young adults and health care provider perceptions of the information provided on social media, focusing on trust and honesty in advertising and promoting drug substances during the COVID-19 pandemic.

## Methods

The participants in the workshops were purposively selected and recruited from local community youth groups and healthcare facilities. Three workshops were conducted on people’s understanding of drug advertising and promotion. Two workshops consisted of ten young adults aged 18-35, the third workshop was conducted with four health personnel: one pharmacist, one traditional practitioner and two healthcare practitioner that participated in prescribing and dispensing medications during the COVID-19 pandemic. The first workshop explored knowledge and perceptions regarding drug purposes, risks, benefits and treatment options included in drug advertisements. The second workshop was conducted with the same of young adults’ group, discussing ways to improve drug risk awareness and understanding for all age groups on the problems of misinformation and malpractice in advertising of drugs on various media platforms. During the workshop with the health care providers, we focused discussing the promotion, advertisement, and publicity of medication on social media according to the national health policies and regulations.

An experienced female research assistant (JN), collected the data using a semi-structured topic guide. Ethical approval was sought from the Biomedical Ethics Committee; BREC/00003859/2022 and granted on the 3 ^rd^ of April 2022. The workshops were conducted face to face in IsiZulu (the main local language) at the youth centres and one at the research site. Each workshop lasted between 45-60 minutes. Informed consent was sought before each workshop when the participants were briefed on the purpose of the study and invited to provide written informed consent. Participants signed the written informed consent before all the data was collected. All data collection activities were digitally recorded, transcribed, and translated into English and stored in a password protected data server. Debriefing meetings were conducted after each workshop between the interviewers and the lead author of this paper using the field notes that the interviewer wrote during the interviews. The debrief meetings aimed at improving probing and providing clarity on the emerging themes.

Data analysis was done manually, led by the first author (RSC), and the research assistant (JN) who collected the data. The coding framework was developed by the team based on a sample of transcripts which the entire team read and used to identify emerging codes. Deductive coding was done using a predefined set of codes developed from the coding framework. We read through the data and assigned excerpts to codes. Themes were later created from the patterns deduced from the analysis. Three main themes were created namely: responsible drug advertising, risks associated with drug and substance promotions on social media, and recommendations on drug risk awareness during COVID-19.

## Results

### Responsible drug advertising

All the young adults who took part in the first two workshops said that social media was an important source of information regarding COVID-19.

*“The truth of the matter is that Facebook is currently the most powerful source of information. On Facebook news feed you will always find COVID-19 related updates, that’s where we find information especially young South African who are very active on Facebook.” (Male, 19 years, workshop 1)*

*“WhatsApp has also become a contributor to obtaining information because there are now hotlines instructing you to send Hi on WhatsApp and then they will update you about everything Covid related for the past 24 hours. It will update you on death cases and so on. That is how I obtain information”. (Male, 25 years workshop 1)*

Sometimes accessing this information was accidental:

*“I mean that I use the internet to do schoolwork and sometimes adverts pop up on the internet and that is how I end up seeing COVID-19 updates.” (Female, 25 years, workshop 1)*

However, while social media platforms were important for obtaining information about COVID-19, one of the health care providers noted that there was often not enough information provided to make informed decisions, because COVID-19 was a new disease.

*“From my point of view most of the people I don’t think they knew about some of the drugs that were supposed to be taken. Like if you got flu, you know which medication to use. So for COVID-19, even the names you can’t even spell the names right. I don’t think they knew about the medication. And there wasn’t a lot of information about everything in general regarding COVID-19 regarding medication, regarding what’s happening. So I feel like people were left out, so they didn’t have full information. I think because it was a new communicable disease.” (Healthcare practitioner, workshop 3)*

This lack of information was a particular point of discussion during the workshop with the health care providers because it may have compromised informed decision-making regarding people’s health. The health care providers highlighted the importance of regulatory bodies in promoting these medicinal products. They did, however, raise concerns about the way this was handled during the pandemic, suggesting that there could have been improvements.

*“[…] in South Africa we have Medicine Act working in hand with the Pharmacy Act as well. So these ones control what is being advertised and one of the requirements from there is, schedule drugs from schedule 3 upwards shouldn’t be advertised. You can inform the public that it is available but you shouldn’t advertise it …” (Pharmacist, workshop 3)*

The health care providers emphasized that certain drugs were too hazardous to be advertised online. While information could be provided about their availability, there was a consensus that these drugs should not have been actively promoted to protect individuals.

The young people mentioned during their workshops that online advertisements did not accurately represent the actual contents of the products. They noted that the primary goal seemed to be profit-driven rather than providing truthful information.

*“The aim of making an advert is to convince people into buying a product. When they are persuaded enough, they will buy it. That is how businesses make income.” (Male, 24 years, workshop 1)*

Another young man (also aged 24 years) corroborated this view: *“The aim of advertisements is to make money. They will make sure that you become attracted to the product enough for you to buy it.” (Male, 24 years, workshop 1).* This emphasis on profit making may conflict with providing honest and accurate information for consumers.

### Risks associated with drug and substance promotions on social media

The young people highlighted the heightened risks associated with drugs promoted on the internet. They noted that certain online advertisements failed to disclose the potential side effects of specific substances, noting that the advertisements ` *only display the positive side of the medicine.”* ( *Male, 24 years, workshop 1*).

The participants raised concerns that by failing to provide honest information about the side effects of the medication, the advertisements withheld crucial information and exposed people to harm.

*“I do not trust the internet[…]we always hear that people had some reactions from taking medication they saw on the internet. You will hear that someone got sick after vaccinating, that is why I do not trust the medicine sold on the internet”. (Male 23 years, Workshop 1)*

The young people highlighted the increased use of traditional medicines, some promoted online, for treating COVID-19 symptoms. They raised concerns about the lack of information provided on traditional medicines which could pose risks for users:

*“No it is not well advertised because they do not give us information on the dosage. Sometimes you mix ingredients to a point where you are afraid to drink it because it looks and tastes so potent and bitter. Sometimes children get severely sick because you find that parent overdosed the child …. Traditional medicine is not well advertised because people do not know when they have used too much or too little”. (Female, 23 years, workshop 1)*

In their workshop the health providers stated that advertising traditional medicine online resulted in lack of accountability since manufacturing and marketing such products may not require approval from regulatory bodies. The pharmacists noted that because traditional medicines are complementary medicines according to the South African Medicines Act these medicines were covered by the guidelines and did require approval. However, one of the traditional healers observed that:

*“If it is my product and I produce that product it is my responsibility to make sure that what is included is indicated there, the dosage is indicated there, everything so that if you take my medication, you know how much you take in a day and you also know the list of the side effects. So I think honestly that is the responsibility of the person that produces that medication. But […] 100% of traditional medicinal products do not comply, do not have those guidelines*.” ( *Traditional healer, workshop 3*)

This indicates a gap in regulatory approval in the protection against harm where traditional and complementary medicines, such as natural herbs, may not comply with the regulations. This can mean that producers can market these products without rigorous safety and efficacy evaluations increasing responsibility to consumers who may be unaware of the potential risks and lack of regulatory oversight.

### Recommendations on drug risk awareness

Recommendations were put forward by participants in all three workshops to ensure that drug promotion information is disseminated and accessed in a fair and equitable way. One young woman (aged 20) observed that:

*“[…]the government should make sure that they provide enough information for people. Although there are adverts but they are not enough … They must take it upon themselves to give us the right information, so we don’t have to listen to random recommendations from [other] people”… (workshop 2)*

Participants highlighted those strategies such as the public directly interacting with health professionals on social media platforms sharing health related information that has scientific evidence can be developed to reduce misinformation.

“ *Make live updates on the internet, a video clip that shows regular updates. Doctors should have their own YouTube channels where they post every day to spread truthful information …”(Female 23 years, workshop 1)*

The young people also suggested that providing such access, via social media, can address inequalities in health information access.

## Discussion

Using community perspectives, we highlighted the importance of trust and honesty in drug promotion on social media during the COVID-19 pandemic in South Africa. People’s trust in social media influenced their personal choices, often regardless of the scientific validity of the promotions. Respecting individuals’ rights to make informed choices about their health and healthcare decisions is essential. In drug promotion, this requires providing accurate and accessible information about medications, potential risks, and alternatives. This approach enables patients to trust the information they receive and actively participate in determining their healthcare needs and selecting the treatment options that are best suited to them. A systematic review done on drug advertisements in medical journals in Africa showed that adequate pharmacological information for advertised drugs is likely to promote and improve rational drug use among consumers. ^11^ Participants in this study expressed concerns because the only information provided to them was on social media and they wondered if it was reliable and evidence based.

Lack of disclosure of side effects in social media advertisements for medications raises potential ethical concerns. This speaks to the principles of Belmont where non-maleficence requires healthcare providers and advertisers to avoid causing harm to individuals. ^12^ By selectively presenting only the positive aspects of a medication while omitting information about potential side effects, ethical obligations to fully inform consumers may have been excluded. ^13^ This omission could lead to harm if individuals make uninformed decisions about their health or experience adverse effects after taking the medication. ^14^

Stakeholders can evaluate the ethical implications of pharmaceutical marketing practices and identify opportunities to enhance transparency, accountability, and responsiveness to community needs. This approach encourages dialogue and collaboration between pharmaceutical companies, healthcare providers, regulators, and community members to ensure that drug advertising promotes ethical principles and contributes to the health and well-being of communities.

Creating social media channels where health care providers and scientists share accurate scientifically proven information creates transparency and cultivates trust and credibility in healthcare communication. ^15, 16^ Increased reliance on social media platforms enhances the reach and speed of public health messaging and the importance of digital communication in managing public health crises globally. ^5, 6^ By allowing healthcare professionals to directly engage with the public, regardless of geographical or socioeconomic barriers, this approach promotes equitable access to reliable healthcare information. However, it is essential that such information adheres to standards for advertising and prioritizes evidence-based content. This ensures that all individuals, regardless of their background or circumstances, have access to accurate and trustworthy information, reducing disparities in healthcare knowledge and decision-making especially in pandemic times.

## Conclusion

This analysis highlights the critical need in respecting individuals’ right to informed choices in drug promotion during the COVID-19 pandemic. The study participants’ concerns about the reliability of social media information and the omission of potential side effects highlight the gaps in ethical considerations in drug promotions on social media. To ensure informed and safe health decisions globally, stakeholders must enhance transparency, accountability, and responsiveness in pharmaceutical marketing. By fostering collaboration between pharmaceutical companies, regulatory bodies, healthcare providers and community members, we can ensure that drug advertising upholds ethical standards and contributes positively to public health.

## Author contributions

All authors directly participated in this study. RSC handled conceptualization, formal analysis, writing—original draft, JN managed data collection, preliminary analysis. JS contributed to supervision, writing-review and editing

## Ethics and consent

Ethical approval for this project was obtained from the University of KwaZulu-Natal Biomedical Research Committee in South Africa (BREC Ref No: BREC/00003859/2022) and granted on the 3 ^rd^ of April 2022. Ethical approval stated that in no way do the requirements for data availability override the right to confidentiality and privacy of individuals or organisations who are the subjects of research. All methods were performed in accordance with the relevant guidelines and regulations.

The workshops were conducted face to face in IsiZulu (the main local language) at the youth centres and one at the research site. Each workshop lasted between 45-60 minutes. Informed consent was sought before each workshop when the participants were briefed on the purpose of the study and invited to provide written informed consent. Participants signed the written informed consent before all the data was collected.

## Paper context

• Main findings

Respecting individuals’ rights to make informed decisions about their health means ensuring that accurate and accessible information is provided about the drugs advertised on social media.

• Added knowledge

Ethical principles that contribute to the health and well-being of communities should be incorporated and regularised more in social media and web-based drug advertising.

• Global health impact for policy and action

Collaboration between pharmaceutical companies, regulatory authorities, healthcare providers, and community stakeholders will ensure that drug advertising adheres to ethical standards, promoting trust and integrity within the healthcare system.

## Data availability

### Underlying data

The interview transcripts cannot be publicly shared because the data contain sensitive information that could potentially identify the participants. The restriction is a guideline set by the ethics committee and included in the informed consent that participants have agreed to. Access to the data is restricted to protect participant confidentiality. Any data access requests must be reviewed and approved by the authors and will only be granted under conditions that ensure participant anonymity. Any data requests can be directed to rujeko.chidawanyika@ahri.org.

### Extended data

Figshare: Extended Data-Community perspectives on drug promotion on social media during the COVID-19 pandemic in KwaZulu-Natal, South Africa. Doi: https://doi.org/10.6084/m9.figshare.27993119.v1. ^17^

This project contains the following extended data:

i. BREC Informed Consent (Drug Advertising) English: this is the voluntary consent participants provide after being fully briefed on the study’s details, as outlined in the research explanation provided to them.
ii. Community Engagement Workshop guide 18_23 years- Interview guide: The tool guide we used during the workshops
iii. Reporting guidelines: we used COREQ checklist

Data are available under the terms of the License: CC0
