# Supplementary material for: Community perspectives on drug promotion on social media during the COVID-19 pandemic in KwaZulu-Natal, South Africa
Source: F1000Res. 2025 Oct 6;14:33. Originally published 2025 Jan 6. [Version 3] doi: 10.12688/f1000research.158535.3 (PMC12572773; doi:10.12688/f1000research.158535.3)
Supplement: Peer review history

# Reviewer report: Reviewer response for version 3

**DOI:** 10.5256/f1000research.188672.r420805

**Published:** 2025-10-29

**Authors:** Nicholas Gibbs

**Affiliations:** 1 Department of Social Sciences, Northumbria University, Newcastle upon Tyne, England, UK

I am satisfied that my comments have been addressed that the article is greatly improved.

Is the work clearly and accurately presented and does it cite the current literature?

Partly

If applicable, is the statistical analysis and its interpretation appropriate?

Not applicable

Are all the source data underlying the results available to ensure full reproducibility?

No source data required

Is the study design appropriate and is the work technically sound?

Yes

Are the conclusions drawn adequately supported by the results?

Yes

Are sufficient details of methods and analysis provided to allow replication by others?

Yes

Reviewer Expertise:

Illicit markets and grey and black market medicines.

I confirm that I have read this submission and believe that I have an appropriate level of expertise to confirm that it is of an acceptable scientific standard.

# Reviewer report: Reviewer response for version 2

**DOI:** 10.5256/f1000research.184077.r394761

**Published:** 2025-09-18

**Authors:** Nicholas Gibbs

**Affiliations:** 1 Department of Social Sciences, Northumbria University, Newcastle upon Tyne, England, UK

It does not appear that any of my recommendations have been actioned in the second version of this paper.

Is the work clearly and accurately presented and does it cite the current literature?

Partly

If applicable, is the statistical analysis and its interpretation appropriate?

Not applicable

Are all the source data underlying the results available to ensure full reproducibility?

No source data required

Is the study design appropriate and is the work technically sound?

Yes

Are the conclusions drawn adequately supported by the results?

Yes

Are sufficient details of methods and analysis provided to allow replication by others?

Yes

Reviewer Expertise:

Illicit markets and grey and black market medicines.

I confirm that I have read this submission and believe that I have an appropriate level of expertise to confirm that it is of an acceptable scientific standard, however I have significant reservations, as outlined above.

# Response

**Published:** 2025-09-23

**Authors:** Rujeko Samanthia Chidawanyika

**Affiliations:** Social Science Core, Africa Health Research Institute, Durban, KwaZulu-Natal, South Africa

**Community perspectives on drug promotion on social media during the COVID-19 pandemic in KwaZulu-Natal, South Africa**

**Reviewer Comments**

 This was an interesting paper that has some significance in the post-pandemic medical landscape. It provides some good context for the work, situating data and analysis in the legal frameworks in South Africa and the use of social media for the dissemination of public health messaging. The piece is coherent and its conclusions reflect the study well. There are a few points, however, that I would raise as necessary to improve the piece.

**Reviewer comment 1**

 Firstly, you do not clarify exactly what medicinal products that you are discussing. Besides a mention of traditional medicines, I was not left aware of the types of medicines and their legal and regulatory status. Were these grey market? Legal? Illegal? 

**Response:**

**We have added text in lines 7-21**

*SAHPRA monitors and regulates the control of medicines in accordance with the Medicines and Related Substances Act 101 of 1965 as amended. Under regulation 42 for advertising and marketing. An “advertisement” according to the Medicines and Related Substances Act refers to “… any written, pictorial, visual or other descriptive matter or verbal statement or reference that  (a) appears in any newspaper, magazine, pamphlet or other publication; (b) is distributed to members of the public; or (c) brought to the notice of members of the public in any manner whatsoever, which is intended to promote the sale of that medicine …”* *(1, 2)* *. In South Africa, medications are categorized or scheduled, and as per legal requirements, manufacturing details can be omitted based on the schedule assigned to the medication* *(1)* *. Medicine schedules are numbers given to pharmaceutical products based on its benefits and its risks. The lower the risk of the product; the lower the number. Unscheduled medicine on the other hand can be purchased at a pharmacy and this medicine has a schedule of 0. Over-the-counter medicine can be purchased at a pharmacy without a prescription and this medicine has a schedule of 0, 1 or 2. Prescription only, controlled substances and strictly controlled substances range from schedule 3-8* *(3)* *.*

**Lines 25-28**

*In this analysis we looked at all legal drugs that were promoted and repurposed for treatment of COVID-19 symptoms. We note that SAHPRA had no guidelines at the time of this study specified for traditional and complementary medicines.*

**Reviewer comment 2**

 Minor one, but there is a missing 's' in the sentence 'wo workshops consisted of ten young adults aged 18-35, the third workshop was conducted with four health personnel: one pharmacist, one traditional practitioner and two healthcare practitioner that participated in prescribing and dispensing medications during the COVID-19 pandemic.'.

**Response:**

 We have edited it in lines 26-31 to read:

*The participants in the workshops were purposively selected and recruited from local community youth groups and healthcare facilities. Three workshops were conducted on people’s understanding of drug advertising and promotion. Two workshops consisted of ten young adults aged 18-35, the third workshop was conducted with four health personnel: one pharmacist, one traditional practitioner and two healthcare practitioner that participated in prescribing and dispensing medications during the COVID-19 pandemic.*

**Reviewer comment 3**

 I think this would do well to engage with the wider scholarly discourse on the health consumer and the political economy of medicines internationally. Notions of both advertising apparatus as well as consumers 'shopping around' for treatment. You could also link this to trust in legitimate medicine and the obfuscation of truth on social media. 

**Response:**

**Thank you for this comment. We have added text in lines 174-185**

*Studies have shown that people's decisions are strongly influenced by the nature and quality of the information they are exposed to and the sources to which they rely on(1). The biomedical knowledge/lack there of, treatment access, and the ready market intersect to shape how individuals engage with medication(2). Medications are commodities  that are part of the production, marketing, and consumption. On social media, consumers are not only positioned as passive recipients of health information, but as active participants that can be able to make choices about self medication and treatments in ways that could contribute to consumer empowerment(3). However there is an increase in possible risks, particularly when misleading advertisements, scientifically unverified remedies, or counterfeit products are promoted alongside the legitimate medications. For users, the task of distinguishing between trustworthy information from misleading or commercially driven outputs shows a larger problem of navigating legitimacy within modern pharmacology on the internet(1).*

**Reviewer comment 4**

 I'd like to see you engage with more literature on the global regulation of medicinal products on social media. Studies have been undertaken in different contexts which can inform your work here. 

**Response:**

**Thank you. We have added text and references in lines 212- 219**

*Studies have indicated the complexities of drug regulation on social media highlighting the critical importance of drug promotions adhering to World Health Organisation and SAHPRA ethical guidelines (7). An improvement in regulatory development is needed with transparent advertising encouraged by policies that show relevance and accuracy of pharmaceutical promotional content on social media, strengthened and enforced (8).*

*A collaborative effort is needed between researchers pharmaceutical companies, health care practitioners, regulators and the public in order to utilise social media as a tool for pharmacovigilance (9).*

**Reviewer comment 5**

 Finally, I think the inclusion of health literacy would benefit the work as well as the digital capital of prospective.

**Response:**

**We thank you for this comment. We have added text and references in lines 231-236**

*Health literacy shapes how communities engage with drug promotion on social media. Health literacy influences the extent to which people can distinguish between marketing and scientific based information , evaluating the credibility of sources, and understanding potential risks or side effects(10). Limited health literacy can increase susceptibility to misinformation, self-medication, or overreliance on unverified online content, reinforcing health inequalities.*

# Reviewer report: Reviewer response for version 2

**DOI:** 10.5256/f1000research.184077.r394762

**Published:** 2025-08-01

**Authors:** Henrik A Hahamyan

**Affiliations:** 1 East Tennessee State University, Johnson City, USA

No comments

Is the work clearly and accurately presented and does it cite the current literature?

Yes

If applicable, is the statistical analysis and its interpretation appropriate?

Not applicable

Are all the source data underlying the results available to ensure full reproducibility?

Partly

Is the study design appropriate and is the work technically sound?

Yes

Are the conclusions drawn adequately supported by the results?

Yes

Are sufficient details of methods and analysis provided to allow replication by others?

Yes

Reviewer Expertise:

Background in social media's impact on the use of performance enhancing drugs

I confirm that I have read this submission and believe that I have an appropriate level of expertise to confirm that it is of an acceptable scientific standard.

# Reviewer report: Reviewer response for version 1

**DOI:** 10.5256/f1000research.174140.r392216

**Published:** 2025-06-16

**Authors:** Henrik A Hahamyan

**Affiliations:** 1 East Tennessee State University, Johnson City, USA

Please provide a full report, including a summary of the article and expanding on your answers to the questions above. In particular, if you answered “no” or “partly” to any of the questions, please give constructive and specific details as to how the authors can address any criticisms. Please indicate clearly which points must be addressed to make the article scientifically sound.

 The study titled “Community perspectives on drug promotion on social media during the COVID-19 pandemic in KwaZulu-Natal, South Africa” intends to discuss and investigate exactly what the title implies. The authors planned separate workshops to identify different perspectives on targeted questions relevant to the topic of drugs discussed on social media. Generally, all three workshops discussed that social media platforms were a source for obtaining information on treatments related to COVID-19 and the unregulated nature led to a lack of accuracy and safety with biased representations.

 The authors illustrate this with a strong study design and contextualize their results well. I understand that we do not have access to all the transcript due to containing sensitive information that could potentially identify the participants; however, it would be useful to include components of more or all responses from the participants, so we could have more information on perspective, while staying within the confines of the consent forms. It may also be helpful to categorize the perspectives in a table which may be more organized and easier to digest. I would also be curious to hear more about what specific, actionable suggestions the authors have to fill in the gaps they have illustrated through their study.

Is the work clearly and accurately presented and does it cite the current literature?

Yes

If applicable, is the statistical analysis and its interpretation appropriate?

Not applicable

Are all the source data underlying the results available to ensure full reproducibility?

Partly

Is the study design appropriate and is the work technically sound?

Yes

Are the conclusions drawn adequately supported by the results?

Yes

Are sufficient details of methods and analysis provided to allow replication by others?

Yes

Reviewer Expertise:

Background in social media's impact on the use of performance enhancing drugs

I confirm that I have read this submission and believe that I have an appropriate level of expertise to confirm that it is of an acceptable scientific standard, however I have significant reservations, as outlined above.

# Reviewer report: Reviewer response for version 1

**DOI:** 10.5256/f1000research.174140.r368335

**Published:** 2025-03-03

**Authors:** Nicholas Gibbs

**Affiliations:** 1 Department of Social Sciences, Northumbria University, Newcastle upon Tyne, England, UK

This was an interesting paper that has some significance in the post-pandemic medical landscape. It provides some good context for the work, situating data and analysis in the legal frameworks in South Africa and the use of social media for the dissemination of public health messaging. The piece is coherent and its conclusions reflect the study well. There are a few points, however, that I would raise as necessary to improve the piece.

 Firstly, you do not clarify exactly what medicinal products that you are discussing. Besides a mention of traditional medicines, I was not left aware of the types of medicines and their legal and regulatory status. Were these grey market? Legal? Illegal? 

 Minor one, but there is a missing 's' in the sentence 'wo workshops consisted of ten young adults aged 18-35, the third workshop was conducted with four health personnel: one pharmacist, one traditional practitioner and two healthcare practitioner that participated in prescribing and dispensing medications during the COVID-19 pandemic.'.

 I think this would would do well to engage with the wider scholarly discourse on the health consumer and the political economy of medicines internationally. Notions of both advertising apparatus as well as consumers 'shopping around' for treatment. You could also link this to trust in legitimate medicine and the obfuscation of truth on social media. 

 I'd like to see you engage with more literature on the global regulation of medicinal products on social media. Studies have been undertaken in different contexts which can inform your work here. 

 Finally, I think the inclusion of health literacy would benefit the work as well as the digital capital of prospective customers.

Is the work clearly and accurately presented and does it cite the current literature?

Partly

If applicable, is the statistical analysis and its interpretation appropriate?

Not applicable

Are all the source data underlying the results available to ensure full reproducibility?

No source data required

Is the study design appropriate and is the work technically sound?

Yes

Are the conclusions drawn adequately supported by the results?

Yes

Are sufficient details of methods and analysis provided to allow replication by others?

Yes

Reviewer Expertise:

Illicit markets and grey and black market medicines.

I confirm that I have read this submission and believe that I have an appropriate level of expertise to confirm that it is of an acceptable scientific standard, however I have significant reservations, as outlined above.
